# Relationship of Acute Phase Reactants and Fat Accumulation during Treatment for Tuberculosis

**DOI:** 10.1155/2011/346295

**Published:** 2011-09-13

**Authors:** Alejandro Sanchez, Colleen Azen, Brenda Jones, Stan Louie, Fred Sattler

**Affiliations:** Division of Infectious Diseases, Department of Medicine, The Keck School of Medicine and the School of Pharmacy of the University of Southern California, IRD Building, Room 432, 2020 Zonal Avenue, Los Angeles, CA 90033, USA

## Abstract

*Background*. Tuberculosis causes inflammation and muscle wasting. We investigated how attenuation of inflammation relates to repletion of body composition during treatment in an underserved population. *Design*. Twenty-four patients (23 to 79 years old) with pulmonary tuberculosis and inflammation (pretreatment albumin = 2.96 ± 0.13 g/dL, C-reactive protein [CRP] = 6.71 ± 1.34 *μ*g/dL, and beta-2-microglobulin = 1.68 ± 0.10 *μ*g/L) were evaluated and had BIA over 24 weeks. *Results*. Weight increased by 3.02 ± 0.81 kg (5.5%; *P* = 0.007) at week 4 and by 8.59 ± 0.97 kg (15.6%; *P* < 0.0001) at week 24. Repletion of body mass was primarily fat, which increased by 2.09 ± 0.52 kg at week 4 and 5.05 ± 0.56 kg at week 24 (*P* = 0.004 and *P* < 0.0001 versus baseline). Fat-free mass (FFM), body cell mass (BCM), and phase angle did not increase until study week 8. Albumin rose to 3.65 ± 0.14 g/dL by week 4 (*P* < 0.001) and slowly increased thereafter. CRP levels declined by *∼*50% at each interval visit. *Conclusions*. During the initial treatment, acute phase reactants returned towards normal. The predominant accrual of fat mass probably reflects ongoing, low levels of inflammation.

## 1. Introduction

Tuberculosis remains one of the most common infections in the world and causes substantial morbidity and mortality. Poor outcomes often relate to severe inflammation and reduced intake of energy and protein that may advance to overt cachexia (protein calorie malnutrition) [1, 2]. As with other systemic inflammatory conditions (e.g., sepsis, burns, massive trauma), proinflammatory cytokines are presumed to orchestrate the release of amino acids from preformed pools in skeletal muscle and breakdown of myofibrillar proteins (acute phase reaction) [3]. These amino acids are transported to the liver and there provide the metabolic fuel for gluconeogenesis and synthesis of new proteins for white cells to combat infection and repair damaged tissue [3], as would occur with tuberculosis. Further, appetite is suppressed during inflammation by TNF*α* [4] and thus ingestion of sufficient nutrients may not occur, even if food is accessible. 

In patients with lean tissue wasting due to chronic hepatitis, renal failure, or AIDS, providing supplemental energy leads to weight gain that is primarily associated with accrual of fat mass with relatively little improvement in lean mass or functional performance [5–8]. It can be speculated that accrual of primary fat mass is the result of ongoing inflammation in those conditions. There is little information available about the quantity and type of body composition repletion during treatment of tuberculosis and how it relates to inflammation [1, 2, 9, 10]. We hypothesized that treatment of tuberculosis, even without supplemental nutrition, would suppress markers of inflammation and improve body mass that would include a substantial portion of new lean tissue, unlike what occurs with nutritional repletion during ongoing inflammation (i.e., primarily fat accrual) in other chronic catabolic disorders. We, therefore, conducted a prospective study to characterize the timing of changes in inflammation as measured by acute phase reactants and their relationship to improvements in body composition during treatment of tuberculosis in an underserved inner city population. Understanding these relationships will be important in developing strategies to substantially augment lean tissue mass, which in general is correlated with enhanced physical function and improved quality of life [11, 12].

## 2. Methods

### 2.1. Study Design

The study was a prospective investigation designed to assess changes in body composition and markers of inflammation during treatment of pulmonary tuberculosis in HIV-negative patients.

### 2.2. Patient Selection

Daily reports of positive sputum smears for acid-fast bacteria generated by the Clinical Microbiology Laboratory of the Los Angeles County-University of Southern California (LAC-USC) Medical Center were used to identify potential study participants. Patients with clinical characteristics and pulmonary radiographs consistent with acute pulmonary tuberculosis were invited to participate. Each signed an informed consent approved by the IRB at USC before enrollment or testing.

### 2.3. Eligibility Criteria

Inclusion criteria required that patients be ≥18 years of age with presence of a positive sputum showing acid-fast bacteria and later confirmed by growth of *Mycobacterium tuberculosis *in culture, less than 96 hours of antituberculosis therapy, negative HIV serology, alanine aminotransferase level less than five times the upper limit of normal, total serum bilirubin ≤2.0 mg/dL, white blood cell count ≥3000 cells/*μ*L, and platelets ≥70,000 cells/*μ*L. Patients were treated with isoniazid, rifampin, pyrazinamide, and ethambutol for the first two months and isoniazid and rifampin thereafter in accordance to the United States Centers for Disease Control & Prevention and American Thoracic Society guidelines.

### 2.4. Outcomes

All participants were examined by a study physician, had body composition measured and laboratory testing at baseline and study weeks 4, 8, 16 and 24. Blood was tested for complete blood counts, serum chemistries, C-reactive protein (CRP), beta-2-microglobulin, and albumin in the LAC-USC Clinical Laboratory.

### 2.5. Body Composition

Body composition measures included height using a stadiometer and weight on the same calibrated scales with participants in underwear and gown. Fat-free mass (FFM), fat mass, body cell mass (BCM), and phase angle were determined by single frequency bioelectrical impedance analysis (BIA; RJL Quantum, Clinton Township, Michigan). For BIA, tests were conducted by a single study nurse certified in electrode placement and body positioning during training at AIDS Clinical Trials Group meetings. Fat, FFM, and BCM were calculated using published equations validated previously in wasted and nutritionally replete patients [13].

### 2.6. Statistical Analysis

For measures of body composition and inflammatory markers (acute phase reactants), patterns of change from baseline over time were analyzed using a repeated measures mixed model for each outcome variable, with weeks as a fixed effect and participants as a random effect, and unstructured covariance. Unless indicated in footnotes, mean values are reported with variance as standard errors, based on estimates generated by the mixed model analyses. Adjusted *P* values were calculated for comparing differences between estimated least squares means at baseline and each of the subsequent time points, or between two adjacent time points. The relationships between total change at 24 weeks in various measures were examined with Pearson correlation coefficients. Two sided *P* values < 0.05 were considered statistically significant.

## 3. Results

### 3.1. Study Participants

Twenty-four consecutive patients meeting eligibility criteria were enrolled. Four participants withdrew from study after the initial baseline examination (two lost to follow up and two refused further blood draws). Diagnosis of *M*. *tuberculosis* was presumed in all participants based on sputum smears showing acid fast bacteria and chest radiographs consistent with tuberculosis. Sputum cultures confirmed the presence of *M. tuberculosis* in each case using standard laboratory methods in a Los Angeles County Public Health reference laboratory. None of the participants received nutritional supplementation during therapy for their tuberculosis. 

Table 1 shows that participants were largely ethnic minorities and their ages ranged from 23 to 79 years. Their BMI was 24.5 ± 0.96 kg/m^2^ and indicated that they were not severely malnourished but their low albumin and elevated CRP and beta-2-microglobulin levels reflected that there was substantive inflammation when treatment was initiated.

### 3.2. Acute Phase Reactants

**Figure 1:**
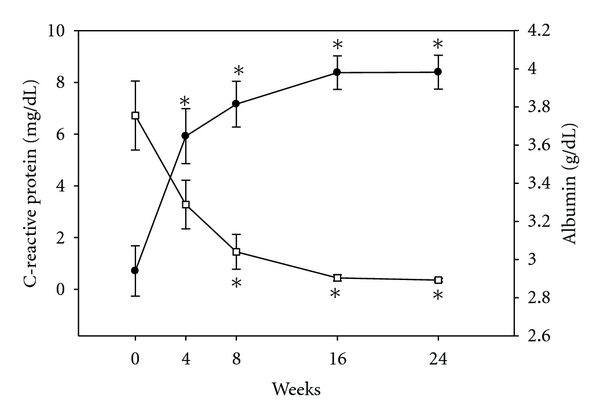
Serum C-reactive protein and albumin during treatment. Figure shows the change C-reactive protein (CRP, open squares) and albumin (filled circles) at each time point compared to baseline. There was an inverse relationship between serum albumin and CRP. *represents significant changes (*P* < 0.05) compared to baseline (week 0); whiskers are standard errors and for CRP at weeks 16 and 24 are small and not be demonstrable on figure.

Pretreatment serum albumin was 2.96 ± 0.13 g/dL, well below the normal range; levels increased briskly to 3.65 ± 0.14 g/dL (*P* < 0.001) by study week 4 and reached a plateau by study week 8 (3.82 ± 0.12 g/dL; *P* = 0.28 versus week 4, Figure 1). There was a reciprocal relationship between serum albumin and CRP. At baseline, CRP levels were elevated (6.71 ± 1.34 *μ*g/dL) but declined by more than 50% at weeks 4, 8, and 16 and reached normal values by weeks 16 and 24 (0.44 ± 0.12 and 0.35 ± 0.067 *μ*g/dL, resp.); levels were nearly different at week 4 (*P* = 0.061) and significantly different than baseline at the weeks 8, 16, and 24 (*P* = 0.004, *P* = 0.0007, and *P* = 0.0007, resp.). Beta-2-microglobulin increased at week 4 and then followed a similar pattern of change to CRP (data not shown since there was a collinear relationship between the two for change over 24 weeks; *r* = 0.70, *P* = 0.002). 

### 3.3. Body Composition

**Figure 2:**
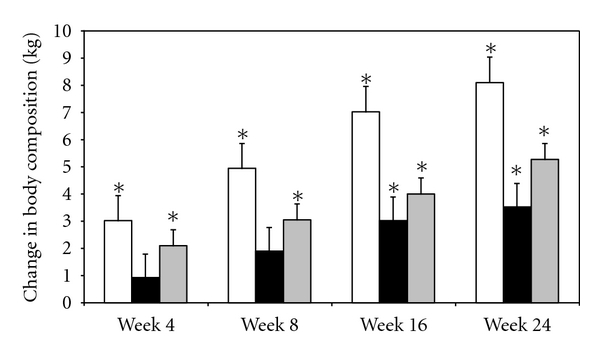
Change in body mass. The change in weight is shown by open bars and fat-free mass by black bars and fat mass by gray bars at each time point compared to baseline. Whiskers represent standard errors. *represent significant change (*P* < 0.05) compared to baseline.

Figure 2 shows absolute change in total body mass, FFM, and fat mass. By study week 4, participants average weight increased by 3.02 ± 0.81 kg (*P* = 0.007) that continued to improve at weeks 8 and 16, and by week 24 was 8.59 ± 0.97 kg (*P* < 0.0001) greater than baseline (Table 2). These improvements in weights represented relative increases of 5.5% to 15.6% from weeks 4 through 24, respectively. FFM also increased continuously over the 24 weeks (Figure 2) but changes from baseline did not reach statistical significance until study weeks 16 and 24 with absolute improvements of 3.02 ± 0.80 kg (*P* = 0.007) and 3.53 ± 0.78 kg (*P* = 0.002), respectively, compared to baseline (Table 2). Fat mass also increased with absolute increments of 2.09 ± 0.52 kg to 5.05 ± 0.56 kg through week 24 with changes at each of the interval visits that were significantly different than baseline (*P* = 0.004 to <0.0001).

**Figure 3:**
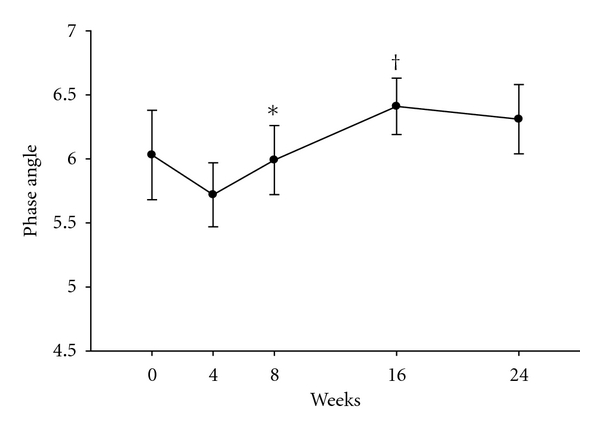
Phase angle during treatment. Average phase angle (shown on *Y* axis) by bioelectrical impedance analysis at each study week. *P = 0.005 compared to week 4. Whiskers represent standard errors. ^†^ *P* = 0.01 compared to week 8.

BCM increased in parallel to FFM, with significant increases over baseline at 16 and 24 weeks of 4.00 ± 0.84 kg and 4.64 ± 0.93 kg (*P* = 0.001 and *P* = 0.0005), respectively (Table 2). Further, low phase angle, an important indicator of malnutrition and poor clinical outcomes, decreased from baseline to week 4 but increased thereafter (Figure 3) with statistically significant improvements at weeks 8 and 16 compared to the prior week (0.27 ± 0.07 and 0.42 ± 0.11, *P* = 0.005 and 0.01, resp.) [14]. 

Table 3 shows the relationships between change in weight, body composition, and acute phase reactants. In brief, as albumin levels increased, this was associated with improvements in total weight and changes in all three acute phases reactants (albumin, CRP, and beta-2-microglobulin) were related to changes in fat mass. Changes in BCM were associated with changes in weight and FFM.

## 4. Discussion

Our study is unique and the first to report changes in acute phase reactants (albumin, CRP, and beta-2-microglobulin) as measures of inflammation and how their levels relate to repletion of body composition components during treatment of tuberculosis. In brief, albumin levels increased quickly by week 4 of treatment and then more slowly thereafter. There were reciprocal decreases in CRP and beta-2-microglobulin, but those occurred somewhat more slowly and did not reach significance until week 8 for CRP and week 24 for beta-2-microglobulin. In this context, total body mass increased briskly by *∼*3 kg at study week 4 and continued to increase each month thereafter. By study week 24, participants had gained *∼*8.6 kg (15.6% increase), thereby demonstrating the effects of treating infection per se and suppressing inflammation on restoring body mass. These robust changes in this underserved population occurred during habitual eating of available foods without nutritional supplementation. 

The rate and magnitude of these improvements in total mass are greater than reported in developing countries (<10% in six months) or in persons coinfected with HIV [5, 15, 16]. It may be that in developing countries there is greater food insecurity and with coinfections (e.g., HIV, parasites), there is continued infection and inflammation, both of which could dampen repletion of body mass. Regardless, demonstrating the feasibility of rapid and substantive weight repletion soon after initiating treatment without nutritional supplementation is of importance since tuberculosis complicated by cachexia is associated with an increased risk of death [17, 18]. 

The composition of the changes in body mass was not as we hypothesized. FFM did not improve significantly until study week 8 with increases just under 2 kg (*∼*40% of increase in total mass) and only modestly increased *∼*3 kg (*∼*43%) at week 16 without further increases at week 24. This was unexpected based on brisk improvement in acute phase reactants in the first two months suggesting that inflammation was quickly attenuated during treatment. However, in the only other report in the modern era that measured components of body composition in patients only infected with tuberculosis, Schwenk and colleagues also showed blunted improvements in FFM with no increases in FFM after one month of treatment with improvements of only 1.5 kg after six months [5]. In our study, fat mass increased significantly by *∼*2 kg at week 4 and then by *∼*1 kg per month until week 24 (*∼*5 kg total increase), and across these time points accounted for almost two-thirds of the increases in total mass in our patients. Similarly, Schwenk and colleagues demonstrated that patients gained 10% in body weight after six months of treatment, and this was almost entirely fat mass [5]. We can only speculate that accrual of largely fat mass in both studies was due to ongoing low-grade inflammation as occurs in other chronic catabolic disorders. In fact, there was still evidence of inflammation during the last 3 months of treatment when levels of CRP and beta-2-microglobulin were just beginning to plateau. 

Macronutrient and total energy intake, which we did not measure, may have affected outcomes as well. During catabolism in sepsis and burns, feeding excess calories has resulted in increasing energy expenditure and futile utilization of fuel with resultant fat deposition without increasing lean mass [19–22]. Our participants were largely underserved and we did not assess quantity or quality of calories that they consumed, which may have been predominantly fat or carbohydrate (not protein) during treatment and attenuation of inflammation. Indeed, even high energy nutritional supplementation administered within two weeks of starting treatment for tuberculosis was associated with primarily accrual of fat mass [9]. During chronic renal failure, patients had to receive protein intakes of about 1.5–2 g/kg/day (twofold or greater than the recommended daily allowance for protein) to achieve positive nitrogen balance [23–26]. We, therefore, speculate that providing optimized dietary protein during treatment of catabolic disorders such as tuberculosis, as with renal failure, is likely to be important in repleting lean tissue, especially BCM, but this remains to be determined [9, 27, 28].

Changes in two other BIA derived parameters may be useful measurements in assessing risk for poor outcomes and monitoring therapy in catabolic disorders such as tuberculosis. BCM when quantified by total body potassium content is reflective of the metabolically active cell fraction of total body mass and relates closely with survival [17, 29]. Improvement in BCM, as occurred in our participants, has been associated with improved outcomes [29–31]. However, BCM, a mathematical derivation from FFM when measured by BIA, was indeed closely linked to changes in weight and FFM in our study, and thus provides little information beyond expected benefits reflective of improvements in FFM. By contrast, the phase angle between resistance and reactance as measured by BIA is a marker of cell integrity and is useful to quantify malnutrition and may be superior to other markers such as BCM, BMI, serum, albumin, cholesterol, and so forth, [14, 17, 32]. Indeed, improvements in phase angle have been associated with better clinical outcomes including survival in patients with HIV [14, 33]. Phase angle increased significantly after an initial decline during therapy in our study. Our participants, who were HIV negative, were alive and free living after six months of therapy. Regardless, improvements in BCM and phase angle after the first month of therapy generally paralleled improvements in body mass, FFM, and acute phase reactants. These findings suggest that BIA may be a useful methodology to monitor body composition, nutritional status, and effects of therapy in more severely ill patients especially in resource limited settings where quantifying lean mass by dual energy *X*-ray absorptiometry or other imaging modalities, access to skilled dietary counseling or anthropometric measurements, and even routine laboratory tests of malnutrition and inflammation are not readily available. 

There are limitations with BIA. FFM is derived mathematically from complex equations that assume the body is a cylinder, and fat mass is the difference of total mass (weight) minus FFM. The methodology should be validated against more rigorous measures in different populations with different baseline morphologic characteristics. Formulas may have to be modified in these populations. In addition, surface electrodes must be properly and reproducibly placed, but training is relatively straight forward and quick. Further, weight needs to be measured in a controlled manner with patients lightly clothed (e.g., gown and underwear) using the same scales periodically calibrated. Finally, height must be carefully determined, which is best done with a stadiometer during proper positioning and should only be measured once since it is squared in formulas for FFM and small variations due to positioning may affect readouts. Regardless, BIA has proven useful in studying and monitoring patients with wasting and should be a cost effective and convenient method to assess nutritional status and body composition in developing countries.

In conclusion, treatment of tuberculosis resulted in prompt and progressive suppression of inflammation with sizable repletion of body mass after only six months of therapy. However, increases in weight were associated primarily with accrual of fat mass despite an apparent rapid attenuation in inflammation based on changes in acute phase reactants. We speculate that to achieve accrual of larger portion of lean tissue and metabolically active body cell mass during treatment may require dietary supplementation with high biologic quality protein or other strategies to improve nutrition or further suppress inflammation. Regardless, findings from this study should have important ramifications for designing future research trials and for treatment of tuberculosis in underserved inner city populations and in developing countries, especially for patients who are cachectic and where there may be food insecurity.

## Figures and Tables

**Table 1:** Baseline characteristics of study participants.

No. of patients	24
Age years*	42 ± 3.06 (23–79)
Male : female	16 : 8
Ethnicity	
Hispanic	20
Non-Hispanic White	1
Non-Hispanic Black	2
Asian-Pacific Islander	1
Total body mass (weight) kg	55.8 ± 2.45 (34.3–73.9)
Body mass index kg/m^2^	24.5 ± 0.96 (16.8–28.8)
Fat-free mass kg	45.7 ± 2.43 (29.77–72.04)
Fat mass kg	9.2 ± 1.78 (−0.8–29.14)
Body cell mass kg	46.8 ± 2.93 (24.3–70.7)
Phase angle	6.03 ± 0.35 (3.3–9.1)
Serum albumin g/dL	2.96 ± 0.13 (1.8–4.5)
C-reactive protein *μ*g/L	6.71 ± 1.34 (1.4–19.4)
Beta-2-microglobulin *μ*g/L	1.68 ± 0.10 (1.0–2.5)

*mean ± standard error (range).

**Table 2:** Changes in body composition during treatment.

Change in body composition	Week 4	Week 8	Week 16	Week 24	*P* value ANOVA
Weight kg	3.02 ± 0.81^∗,a^	4.94 ± 1.08^a,b^	7.03 ± 0.97^b,c^	8.59 ± 0.97^c^	<0.0001
	5.5%**	9.0%	12.8%	15.6%	
Fat-free mass kg	0.93 ± 0.78^a^	1.90 ± 0.95^a, b^	3.02 ± 0.80^b,∗^	3.53 ± 0.78	<0.0001
	2.0%	4.2%	6.6%	7.7%	
Body cell mass kg	0.02 ± 0.95^a^	1.65 ± 1.02^a,b^	4.00 ± 0.84^b,∗^	4.64 ± 0.93	<0.0001
	0.0%	3.5%	8.5%	9.9%	
Fat mass kg	2.09 ± 0.52^∗,a^	3.04 ± 0.50^a^	4.00 ± 0.59^b^	5.05 ± 0.56^b^	<0.0001
	22.7%	33.1%	43.5%	55.0%	

*different at time point compared to baseline (*P* < 0.05); variance (±) is standard error.

**percent change is the ratio of the absolute increase at study week divided by the baseline value ×100.

^a,b,c^ values with same superscript (“a” and “a”) are significantly different (adjusted *P* < 0.05) in pairwise comparisons.

**Table 3:** Relationships of change in weight, body composition, and acute phase reactants.

	Change in weight*		Change in fat-free mass		Change in fat mass	
	Pearson *R* value	*P* value	Pearson *R* value	*P* value	Pearson *R* value	*P* value
Change in albumin*	0.58	0.01	0.21	0.42	0.59	0.01
Change in C-reactive protein	−0.23	0.41	0.19	0.49	−0.58	0.02
Change in beta-2-microglobulin	−0.03	0.93	0.36	0.19	−0.50	0.051
Change in body cell mass	0.66	0.004	0.73	0.0008	0.06	0.80
Change in phase angle	0.13	0.61	−0.06	0.83	0.27	0.29

*changes in body composition parameters and acute phase reactants from baseline to study week 24.
